# President’s Address

**Published:** 1902-07-15

**Authors:** C. H. Oakman


THE
### DENTAL REGISTER.

Vol. LVI. July 15, 1902. No. 7.

### PRESIDENT'S ADDRESS.

BY DR. C. H. OAKMAN.

Read at the Michigan State Dental Association, Grand Rapids, June 9, 1902.

*To the Members of the Michigan Dental Association:* —In presenting to you the President's annual address, I do not presume to offer anything original, but to reiterate a few points which to my mind are most important. This association was organized forty-six years ago with ten charter members. To-day we have 136 active members, quite an increase, but when we consider the lapse of time, we must confess that the society has not grown as rapidly as it should have done.

This association should not rest content on its past efforts, for we have hundreds of good dentists who ought to be enrolled on our books, and become active workers in the upbuilding of this association, and this can only be accomplished by the united efforts of every member of this body. Scores of young graduates are locating in this State every year, to practice the profession of dentistry, and if these young men were given the right hand of fellowship and invited to join us, we would at least have done our part to start them in the right way.

If the teachers in all our dental colleges would endeavor to impress on the minds of the students the necessity for early affiliation with some dental society, the battle would be half won. This would be in the nature of home missionary work. When dentists *realize* that it is the profession which will give them their reputations as dentists, and not the public, then we can look for a new activity in our society work. The petty jealousies which have been so marked in the past, are nearly obliterated, and they will surely cease entirely, when we have a better understanding of one another. This can be best accomplished by meeting frequently in society work and touching elbows at least once a year.

I would suggest the appointing of a body of practicing dentists, by the governor of the State, or mayors of our cities, or health board, to examine the children's teeth in our public and State institutions. As a result, the chances for saving the first permanent teeth would be greatly enhanced. The first molar is well called the keystone of the arch. Hundreds of cases of malocclusion due to its untimely removal are too apparent for me to dwell upon. A great deal has been written on this important subject, and I hope much more will follow, until we have a proper realization of the value of early attention to this important tooth.

As there are only about twenty percent of the people of the country, who now have their teeth cared for by the dentist, it would seem that the early training of the children would change the statistics very materially within the next ten years.

New methods of practice are developing so rapidly in all branches of dentistry, that it is now almost impossible for one person to become expert in all of them. How are we going to obviate this?

I would suggest the encouragement of those who have ability in any particular department, to enter that as a special field, for the time has come when the specialist in dentistry is as essential as the specialist in medicine. This will no doubt raise the professional standards, as it will raise up a body of experts who will increase the standard of excellence in their respective specialties.

There is no profession which has made greater progress in recent years, than dentistry. When we look backward to the last decade and note the progress made in oral surgery, bacteriology, prosthetic art, the application of the X-ray and other modern science, etc., many appliances and instruments, and the numerous new pharmaceutical preparations for the alleviation of pain, which are particularly suited, if not essential to modern dental practice, we can not fail to be impressed with the vast progress made.

The progress made in porcelain art is indeed commendable, but as yet, it is only the few experts who can produce the degree of success to which we all aspire.

The good work done by the State board is gratifying. There is yet much to be done in bringing illegal practitioners to account. This can not be accomplished without the co-operation of every legalized practitioner within the confines of our State. The State Board of Examiners are public servants, and as such are ever ready to unite in lending their aid in the prosecution of every case of violation of the law.

The renewing of acquaintances, the reading of papers, and the study of clinics, must necessarily produce food for the serious consideration of every dentist in attendance, and we trust that all may be free to give as well as to receive.

Two years ago the President of this Association, in his annual address, dwelt upon the necessity for united effort in behalf of this Association. It is my earnest appeal to each and every member, to do something tangible for this Association. Not only is your presence necessary, you should try to induce your neighboring practitioners to attend, providing they are upright and honorable men. What a power of strength this body would be if each of its members should bring in another member within the next year. It would be encouragement to us all, We should endeavor with renewed vigor to follow the ideals conceived and practiced by the founders of our Association, and endeavor by all means to promote the art and science of dentistry. The infusion of new blood is a necessity for the maintenance of this Association, and the accomplishment of its worthy and desirable ends.

It has been for a long time the concensus of opinion held by our members, who have held executive offices, that our present constitution was not sufficiently up-to-date to enable us to provide meetings of sufficient interest to the general practitioner, and to encourage the larger participation of our members, which is calculated to foster interest and enthusiasm. Two years ago a committee was appointed to make such revision of our Constitution, as might serve to correct this condition of affairs. This committee has carefully gone over the old constitution, and after comparing the constitutions of various other state organizations, has culled from them their strongest features, and incorporated them in a report which has been printed and mailed to every member of this Association, with the request that careful perusal might be had before coming to its consideration and adoption by this meeting. The radical departure recommended by your committee of delegating, almost entirely, the executive business of the body to the Board of Directors, may at first sight seem to you unwise, but it has been under trial by the Minnesota, Illinois, and Ohio State Societies for several years, and works most happily, and nothing but the highest praise was reported to your committee of this method. It delegates to a trusted few the common business interests which have little professional concern or value, and gives more time for the free discussion of the more serious matters of practical value. The other departure of electing a Board of Trustees, for a term of three years, is in the interest of a more carefully devised program, and a closer watch over the general professional concerns of a legal nature, All who have had to do with the matter of providing a program for a dental convention of such magnitude as this is, or deserves to be— and which we trust will be in the very near future,— will appreciate the wisdom of this suggestion of our committee. The idea is, that a man placed in charge of such a matter for a term of three years, will not only learn how, but if he have any proper conception of the honor conferred, and the sacredness of his trust, he will begin early to formulate his program, and provide such entertainment as shall not fail to allure even the careless and unappreciative practitioner away from his shop a few days each year. We regard the "program" and "clinic" trustees as great inventions, and wonder why we did not long ago discover them. The "legal" trustee is none the less important. It is not designed that this officer shall be a dental prosecutor of illegal practitioners etc., but that he shall be posted about all matter having a legal bearing upon our profession, and shall keep the profession at all times advised of threatening danger to our laws, or the necessity for new enactments. He shall also keep us in close touch with our State Board of Examiners, and all other transactions of a legal character of professional interest; a kind of walking delegate, as it were in law matters, who shall be in official position to rally his cohorts in time of danger. This amended constitution will come up for your considera tion at an early part of this session, and we urge you to give it earnest and careful consideration, as it is probably the most important business of this meeting.

Another matter of great importance, which must be decided at this meeting, is the question as to what we shall do in regard to the holding of another "Tri-State" convention in 1904. I need not here rehearse the delightful and profitable gatherings which have resulted from these remarkable conventions. As we were originators, and first hosts, it devolves upon us to decide if we desire to continue these fraternal and social amenities any further. Other states stand ready to join us, or rather to accept our hospitality, should we care to enlarge our social relations. These conventions as you well know, are a considerable tax on our financial resources but if taken in time, and by careful management, the expense may be easily taken care of, and need not exceed the abilities of this organization, and its affiliated local organizations.

